# Provider perceptions of barriers to the emergency use of tPA for Acute Ischemic Stroke: A qualitative study

**DOI:** 10.1186/1471-227X-11-5

**Published:** 2011-05-06

**Authors:** William J Meurer, Jennifer J Majersik, Shirley M Frederiksen, Allison M Kade, Annette M Sandretto, Phillip A Scott

**Affiliations:** 1 Department of Emergency Medicine, University of Michigan, Ann Arbor, Michigan, USA; 2 Department of Neurology, University of Michigan, Ann Arbor, Michigan, USA; 3 Department of Neurology, University of Utah, Salt Lake City, Utah, USA

## Abstract

**Background:**

Only 1-3% of ischemic stroke patients receive thrombolytic therapy. Provider barriers to adhering with guidelines recommending tPA delivery in acute stroke are not well known. The main objective of this study was to describe barriers to thrombolytic use in acute stroke care.

**Methods:**

Twenty-four hospitals were randomly selected and matched into 12 pairs. Barrier assessment occurred at intervention sites only, and utilized focus groups and structured interviews. A pre-specified taxonomy was employed to characterize barriers. Two investigators independently assigned themes to transcribed responses. Seven facilitators (three emergency physicians, two nurses, and two study coordinators) conducted focus groups and interviews of emergency physicians (65), nurses (62), neurologists (15), radiologists (12), hospital administrators (12), and three others (hospitalists and pharmacist).

**Results:**

The following themes represented the most important external barriers: environmental and patient factors. Important barriers internal to the clinician included familiarity with and motivation to adhere to the guidelines, lack of self-efficacy and outcome expectancy. The following themes were not substantial barriers: lack of awareness of the existence of acute stroke guidelines, presence of conflicting guidelines, and lack of agreement with the guidelines.

**Conclusions:**

Healthcare providers perceive environmental and patient-related factors as the primary barriers to adherence with acute stroke treatment guidelines. Interventions focused on increasing physician familiarity with and motivation to follow guidelines may be of highest yield in improving adherence. Improving self-efficacy in performing guideline concordant care may also be useful.

**Trial Registration:**

ClinicalTrials.gov identifier: NCT00349479

## Background

Ischemic stroke is a devastating disease, affecting approximately 600,000 adults in the U.S. every year, leaving many survivors with significant functional limitations[1]. Intravenous administration of tissue plasminogen activator (tPA) is recommended by American Heart Association (AHA) guidelines for the early treatment of acute ischemic stroke[2,3]. However, only 1% to 3% of all ischemic stroke patients in community settings receive thrombolytic therapy; this is estimated to be about half of those eligible[4,5]. This low rate suggests numerous barriers exist at both the provider and institutional levels[6].

A large proportion of patients are excluded from treatment due to factors outside of physician control, such as delayed presentation to the hospital. In spite of this, provider-specific barriers remain a significant determinant of low treatment rates[4,6]. Previously it has been shown that professional education can improve treatment rates in stroke[7]. However, for the educational effort to be successful it is critical that the effort is tailored to the targeted populations of providers[8].

The INcreasing Stroke Treatment through INterventional behavioral Change Tactics (INSTINCT) trial is a cluster randomized, controlled trial aimed at increasing appropriate tPA use in ischemic stroke by first determining hospital-specific barriers and then providing targeted, professional educational interventions. Barriers were determined using a partial grounded theory method, whereby qualitative data obtained through focus group discussions is coded into themes using a previously-described taxonomy[9]. Qualitative methods are uniquely suited to develop understanding of complex situations that are difficult to measure quantitatively[10]. The milieu of clinician attitudes, institutional practices, and hospital resources involved in emergency stroke care in the community is a prime example of such a setting for which qualitative methods may provide important insights. Our primary objective was to describe the qualitatively-derived barriers to clinician compliance with guidelines recommending the use of tPA in appropriate patients as discovered in the barrier assessment phase of INSTINCT.

## Methods

### Ethics Statement

The protocol was approved by the University of Michigan Institutional Review Board (IRBMED) and all relevant site IRBs. Written informed consent was obtained from all participants in focus groups and interviews.

### INSTINCT Trial Overview

**Figure 1:**
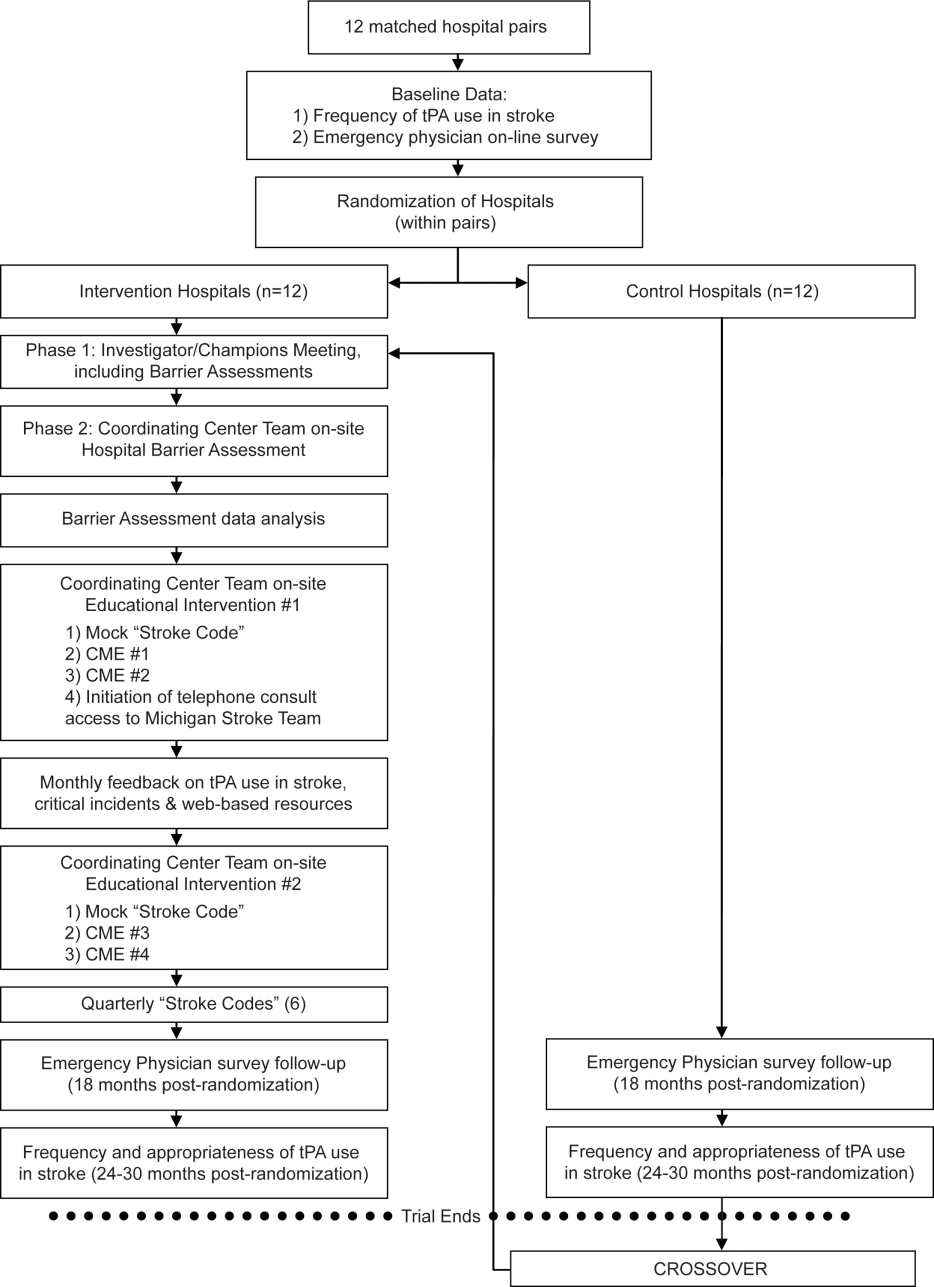
**Overview of INSTINCT trial**. Process of barrier assessments and interventions at INSTINCT hospitals.

The INSTINCT trial is evaluating the hypothesis that initial barrier assessment focused on tPA use in stroke followed by targeted, interactive educational interventions will increase appropriate tPA use[11]. These educational initiatives were planned to be specifically tailored to the needs of each site. A schematic of the INSTINCT trial is depicted in Figure 1. After site selection and randomization, an initial period of barrier assessment was conducted which involved focus groups, interviews, and surveys. The results of the barrier assessments were then used to tailor site-specific continuing medical education (CME) lectures to the most important barriers that participants reported. Additional interventions to improve stroke care occurred concurrently and included assistance with clinical protocol development, 24-7 telephone access to the University of Michigan acute stroke team, mock stroke codes, and targeted messaging. Examples of targeted messaging include informing participants of their site's progress and the overall performance of other sites within INSTINCT and critical incident debriefing, where a physician from the clinical coordinating center contacted local physicians to discuss specific instances of deviations from American Stroke Association guidelines or treatment complications. Appropriate use of tPA and total number of stroke patients were tracked at each hospital throughout the study period and served as the primary method by which the INSTINCT intervention was measured. Finally, the INSTINCT trial required the recruitment of a local stroke champion at each site to serve as the local principal investigator and to act as a liaison between the INSTINCT trial clinical coordinating center and the health care providers at each site.

### Study Setting

Twenty-four hospitals were randomly selected from the population of Michigan acute care hospitals and matched into 12 pairs based on emergency department volume and number of stroke patients (See Figure 1). Hospitals that were established academic comprehensive stroke centers were excluded. Primary stroke centers were not excluded, but were relatively uncommon in the hospital sample at the time of randomization. Each pair contained an intervention site and a control site, randomly assigned. Intervention group hospitals were 25% urban with a total aggregate annual emergency department volume of 397,193 in 2007.

### Rationale for qualitative inquiry

An overall goal of the qualitative inquiry was to design a process which would complement existing quality improvement programs, such as Get With the Guidelines (GWTG)- Stroke[12]. While GWTG-Stroke provides important tools for measuring progress, it is limited in its specific ability to encourage clinicians to comply with guidelines recommending intravenous tPA to eligible stroke patients. This is of particular importance in the United States, where emergency physicians (EPs) are typically the frontline of acute stroke care. In most U.S. practice settings, immediate access to a neurologist or stroke specialist does not exist[13]. Many decisions regarding stroke treatment, up to and including thrombolytic use, are made by EPs. Even in settings with access to acute stroke teams, the emergency care providers (physicians and nurses) need to recognize that the patient is having a stroke and alert the stroke team. In both instances, clinician beliefs about the relative efficacy of stroke thrombolysis, physician expertise, past experience, and concern about adverse effects influence the efficiency and overall tone of the decision-making process. Thus, the initial relationship at the bedside between clinician and decision maker (patient or family member) considering thrombolysis for stroke is both complex and ill-defined[14]. In a large proportion of community hospitals in the United States this role is most commonly filled by EPs.

### Overview of data collection process

The qualitative data collection and analysis methods have been described in detail previously and are summarized below[15]. During design, data collection, and analysis, we adhered to the consolidated criteria for reporting qualitative research (COREQ) when possible as outlined in Table 1[16]. The qualitative inquiry occurred in two phases. Phase 1 consisted of exploratory focus groups that were conducted at a central meeting of stroke champions and stakeholders from each of the intervention sites; the results of these discussions were used to refine discussion guides for phase 2. During the phase 2 barrier assessment process, both focus groups and one-on-one interviews were conducted by the INSTINCT trial team at each of the 12 intervention hospitals.

**Table 1:** COREQ Checklist

1. Interviewer/facilitator	WJM, AMS, SMF, and PAS conducted the interviews and focus groups - with exception of three focus groups at Champions Meeting (phase 1) facilitated by individuals in the Acknowledgements.
2. Credentials	Three physicians, two nursing study coordinators, a human subjects protection coordinator, and a geriatric emergency medicine study coordinator performed interviews and focus groups.

3. Occupation	All facilitators were employees of the University of Michigan. PAS, AMS, SMF received salary support from the cited NIH grant.

4. Gender	All physician facilitators were male. All other facilitators were female.

5. Experience and training	Half of the facilitators had prior focus group experience. A marketing professional with experience in qualitative research provided in-person training in focus group conduction and analysis to WJM, AMS, SMF, and PAS in February of 2007.

6. Relationship established	A prior relationship between the facilitator and participants did not exist in most cases. PAS conducted recruitment of site Principal Investigators; AMS and SMF performed site monitoring and in some cases had a prior relationship with participants.

7. Participant knowledge of the interviewer	Prior to any data collection, we provided all potential participants with an overview of the INSTINCT study prior to signing of the informed consent. The facilitator provided a brief introduction prior to beginning an interview or focus group.

8. Interviewer characteristics	PAS, WJM, AMS, and SMF are in favor of tPA use when local conditions ensure that it can be delivered in accordance with published guidelines. PAS is a co-author of the 2007 American Stroke Association/American Heart Association guidelines for the Early Management of Acute Ischemic Stroke. The other facilitators recognized in the acknowledgments section have no specific opinion for or against the treatment.

Domain 2: Study Design	

9. Methodologic orientation and theory	We used the taxonomy described by Cabana to categorize barriers to behavioral change from the perspective of the physician.^8 ^To further describe our findings, we used grounded theory to inductively derive additional themes that characterized the transcripts.

10. Sampling	All emergency physicians and nurses at each site were invited to participate in the focus groups. Representatives from administration, radiology and neurology were approached based on the recommendations of the local site PIs, thus this was purposive sampling.

11. Method of approach	Participants were identified by the local site investigators and their coordinators.

12. Sample size	Our goal was to achieve participation from several stakeholders in acute stroke care at each site. Our sampling method allowed for prioritization and customization of targeted educational interventions at each site; this was the main objective of the qualitative analysis.

13. Non-participation	One physician decided not to participate in a focus group after the informed consent was explained as he was concerned that his participation in another stroke study represented a possible conflict.

14. Setting of data collection	We used a large conference room at a hotel during the initial site investigator meeting. At each of the participating hospitals, we utilized ED conference rooms, offices, and classrooms.

15. Presence of non-participants	Our protocol did not allow non participants. Any non-participants were immediately asked to leave if they entered the room where an interview or focus group took place.

16. Description of sample	Our protocol only allowed for collection of occupation. Demographic characteristics of the participants were not collected.

17. Interview guide	This was pilot tested during phase 1 and improved for phase 2. The focus group discussion guide is available as additional file 2 (appendix_focus_group_script.doc).

18. Repeat interviews	Our protocol did not specifically allow for this. In some cases, participants from the initial site investigator meeting also participated at the site barrier assessment focus groups or interviews. As the transcripts were de-identified to protect the subjects, the exact number of times this occurred is unavailable, but is approximately in the 5-10 range.

19. Audio/visual recording	Digital audio recordings were made and transcribed verbatim.

20. Field notes	Field notes were not taken in real time. Digital recordings were rapidly reviewed upon the return of the study team to the clinical coordinating center. In the event of recording failure, the facilitator created field notes based on memory and the discussion guide. Recording failure occurred in only two interviews and in no focus groups.

21. Duration	The initial site investigator meeting focus groups were about 90 minutes. The on-site focus groups were approximately 45 minutes. The individual interviews lasted 20 - 30 minutes.

22. Data saturation	While achieving data saturation is an important aspect of qualitative research, our design did not allow for repeat site visits.

23. Transcripts returned	We did not return transcripts to participants for comment and/or correction. Our protocol did not allow for this and the transcripts had personal identifiers removed to protect the participants in the event of a security breach.

Domain 3: Analysis and Findings	

24. Number of data coder:	WJM and JJM performed all coding.

25. Derivation of coding tree:	The coding tree used for initial assignment into the 9 major themes is adapted from Cabana and described in the methods.

26. Derivation of themes	Major themes were derived in advance; minor themes were derived from the data inductively.

27. Software	NVivo 7 was used for data analysis and management.

28. Did participants provide feedback on the findings	At educational interventions later, although protocol did not allow for collection of this data.

29. Quotations presented	See results section

30. Data and findings consistent (was there consistency between the data presented and the findings)	Questions 30-32 of COREQ address the evaluation of the findings of a qualitative study and are intended for readers of qualitative research. They are included here for completeness. We have attempted to present our findings in this work clearly in a manner that was consistent with the data collected.
	
31. Clarity of major themes	
	
32. Clarity of minor themes	

Methods and reporting according to COREQ statement.

### Participants

#### Characteristics of participants

There were 30 participants in the six initial focus groups (phase 1): 10 EPs, 15 nurses, 3 neurologists, 1 hospitalist, and 1 pharmacist. Focus group composition during phase 1 was mixed by site and occupation and the groups ran concurrently. In phase 2, two focus groups were conducted at each of the 12 intervention sites, one of EPs and one of primarily emergency department nursing staff. A total of 55 EPs and 48 nurses participated in phase 2 focus groups. Additionally, one-on-one structured interviews were conducted with a neurologist, an administrator, and a radiologist at each intervention site. Focus group participants were recruited by the local principal investigator from each site. Participants with disparate opinions and past experience were sought to enhance the diversity of responses. The demographics of these participants were not collected to protect anonymity.

#### Data Acquisition

The focus group discussion guide was developed with a professional focus group consultant. It is included in Additional file 1 (appendix_focus_group_script.doc). All focus groups and interviews were digitally recorded and transcribed verbatim.

#### Thematic Analysis

A pre-specified taxonomy was employed to characterize major barriers to clinical guideline adherence[9]. Barriers were broadly characterized as internal or external. External barriers were defined to describe issues inhibiting guideline adherence outside the direct control of physicians. Internal barriers were defined as those barriers that are directly related to individual physician knowledge and attitudes. Two investigators (JJM, WJM) independently coded the transcripts into themes using NVIVO 7 software (QSR International). The coding guide is presented in Table 2, with the comprehensive coding guide used by the investigators provided in Additional File 2 (Appendix_coding_guide_v1.3.doc). The pre-specified major themes were utilized to optimize the process by which the major barriers were categorized and ranked to prioritize the CME educational interventions at each site. Specific textual content that provided insights into the types of barriers at each site was used in the design of the CME lectures. As an example, if a participant identified that radiologists were not routinely notified that a head CT involved a tPA-eligible patient, the CME lecture at that site could contain specific advice on optimizing communication between clinicians and radiologists.

**Table 2:** Coding Guide and Barrier Definitions

Lack of Guideline Agreement	This barrier is coded when the text relates to the respondent not agreeing with the guidelines. This can include but is not limited to personal interpretation of the evidence, applicability to specific patients, and lack of confidence in the guideline developer or the process by which the guideline was developed. Similarly, this barrier is coded if the respondent cites national or local opinion leaders who disagree with this guideline.
	
	This barrier should also be coded if a general lack of agreement with guidelines in general (i.e. "too cookbook") is observed.
	
	This category also includes being too liberal in treatment despite the presence of absolute contraindications to treatment (such as time.)
Lack of Guideline Awareness	This barrier is coded when physicians are not aware of the existence of guidelines for acute stroke care.
	
	It is also appropriate to code this barrier in cases when the lack of awareness is in other members of the patient care team (i.e. inpatient team being unaware of guidelines regarding blood pressure management); in such an instance, it may also be appropriate to code as an environmental barrier if it appears to be a reflection of institutional politics or common practice.
	
	This code does **NOT **include not knowing about the existence of stroke scales.

Lack of Guideline Familiarity	This barrier is coded when there is a lack of knowledge of guideline contents or the inability to properly access or apply the guideline. This includes overuse or desire for overuse of tPA outside of the guidelines (i.e. feeling that a strict time window is not necessary to ensure safe treatment).
	
	This category is not meant to reflect a lack of familiarity with emergency care in general or with stroke patients in general. However, if a respondent cites that they only see one eligible stroke patient every 5 years and do not recall all of the inclusion and exclusion criteria, this barrier should be coded.
	
	This barrier is coded for a reluctance to treat those at the extremes of age and at the extremes of severity since the guidelines which do not include these clinical findings as contraindications (other than very low severity and age < 18 years.)
	
	Physicians and nurses who fail to recognize stroke symptoms are included here (but not EMS providers, which are considered external to the ED and are thus coded as an Environmental Factor.).

Lack of Outcome Expectancy	The physician believes that the performance of the guideline will not lead to the desired outcome or there is a prominent, stated fear of a bad outcome.

Lack of Self Efficacy	The respondent believes that they cannot perform the guideline recommendation correctly. This may be a reflection of personal experience or available resources. (However, a lack of available resources generally should be coded as an External Barrier - Environmental Factor.)
	
	This can also reflect a situation in which the physician or nurse feels unable to treat the patient effectively with the tools they are given (i.e. a vague reading from radiology makes it hard to confidently offer tPA).

Lack of Motivation	Inertia can be a powerful force. This barrier should be coded when the discussion includes the difficulty in changing clinician habit and routines.
	
	This should also be coded when it appears that there is "reluctance" to treat. Willingness to treat, whether physicians "like tPA" or not, and other concepts relating to physician perception reflect a lack of motivation to comply with the guideline.

External Barriers - Environmental Factors	This is a large category. It encompasses the environment in which care is delivered. It includes lack of resources, institutional hurdles, lack of consultants, lack of reimbursement, and, of special importance in acute stroke care, liability. In acute stroke care, pre-hospital, triage and overcrowding issues also fall into this category.
	
	Issues surrounding patient geography (e.g. difficulty in EMS covering rural areas) generally should be included here.
	
	Inpatient floor and nursing home issues are also included under Environmental Factors.

External Barriers - Patient (and Family) Factors	There are many patient and family factors. Some examples:
	
	Patients may fail to recognize stroke symptoms or to present in a timely fashion.
	
	Family preferences to receive or not receive tPA and difficulty in finding family for the consent process are Patient Factors.
	
	Difficulty in communication due to language barriers.
	
	Delayed presentation due to geography would usually be an environmental factor; however if the family decides to drive the patient instead of activating EMS this would qualify as a Patient Factor.
	
	If the patient chooses an inappropriate level of care for their symptoms (i.e. presenting to an urgent care center with a dense hemiparesis) that would qualify as a Patient Factor; however if EMS and the urgent care center cannot promptly move that patient to a facility with an appropriate level of care that would then generally be an Environmental Factor.

External Barriers - Guideline Factors	The characteristics of the guideline itself can present a barrier. The presence of contradictory guidelines or "position statements" would fall into this category. This includes lack of confidence in the guideline, the body or bodies which create the guideline, and the guideline development process. If the guidelines are not felt to be clear, this would also be in this category.

Major barrier categories and instructions used by investigators when coding the interview and focus group data.

Responses from participants were coded into nine major themes. The three external barriers were environmental factors (e.g., availability of intensive care units, ED crowding, pharmacy or radiology), patient factors (e.g., failure to recognize symptoms, preference to arrive via car instead of ambulance), and guideline factors (issues with the structure or content of guidelines in general). The six internal barriers were the lack of familiarity, agreement, awareness, motivation, outcome expectancy, or self-efficacy. Each paragraph (the coding unit) was coded for all themes found; thus each paragraph could be assigned zero to nine themes. See Table 2 for a detailed description of all of the major coding themes.

Major themes were derived in advance of data collection. After completion of phase 1, the two coders independently used the phase 1 data to inductively derive minor themes, including the various aspects of acute stroke presentation and treatment, conceptual models of acute stroke presentation, and the overall process of stroke onset to outcome. These minor themes were then coded for both phase 1 and 2 data for the development of the site-specific educational interventions. Barriers were also related to the various phases of acute stroke presentation and treatment. External barriers were related to the conceptual models of the acute stroke presentation. Barriers were related to the points in the overall process from stroke onset to outcome.

#### Timeline

Phase 1 of the barrier assessments occurred at the initial site investigators' meeting on 3/26/2007. Phase 2 of the barrier assessments was conducted at each of the intervention hospitals from 6/12/2007 to 10/05/2007. The thematic analysis occurred from July to October 2007 and was used to design and prioritize educational interventions for the trial. The short lead time from barrier assessment to intervention was the rationale for the semi-quantitative approach (relative barrier proportions) that was utilized to determine the most discussed barriers from each site.

## Results

**Figure 2:**
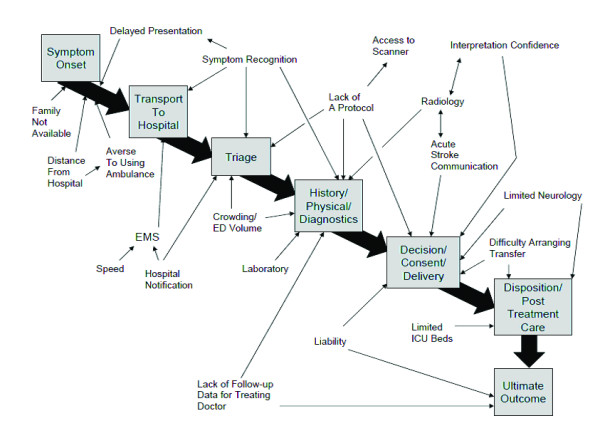
**Relationship of acute stroke care process to barriers external to the emergency physician**. The pathway shows the process a patient would go through when presenting with an acute stroke. The relationship of the identified external barriers to each point on this pathway is demonstrated here.

Since the external barriers of environmental and patient factors comprised most of the cited barriers, sub-categories were inductively derived from these two major themes to better inform the sites during the educational intervention. The derived subcategory themes of barriers external to the EP are described in Table 3 and provided within the framework of acute stroke presentation in Figure 2. The temporal process of stroke occurrence, presentation, treatment and recovery that leads to the final outcome is shown.

**Table 3:** Sub Categories of Identified Barriers External to the Individual Provider

Environmental Factors	n	Patient Factors	n
Radiology	195	Delayed presentation	92

-Access to scanner	43	Symptom recognition - patients/family	50

-Acute stroke communication	79	Family issues	15

-Interpretation confidence	51	Language	5

Limited neurology	108	Adverse to taking ambulance	5

ED overcrowding	54	Demand for tPA	4

Laboratory	49	Age of population	2

EMS	46		

-Hospital notification	4		

-Speed	9		

-Symptom recognition	16		

Pharmacy and drug Delivery	39		

Liability	39		

Lack of a protocol	38		

Triage	36		

Difficulty arranging for transfer	31		

Inpatient/ICU Bed Availability	26		

Limited neurosurgery	24		

Lack of follow up feedback	18		

Geography	14		

Financial issues	9		

Transfer from clinics	6		

Inaccurate patient weight	4		

External barriers that were categorized into themes. The numbers represent the total number of times these items were discussed as a barrier (within the coding unit or paragraph of a transcript). For example, four separate paragraphs had reference to the inaccurate patient weight barrier. This could have been four separate responses from one participant or four separate responses from four participants. Since each paragraph could be assigned anywhere from zero to nine major themes, and an unlimited number of minor themes there is no total or denominator for the coded responses.

**Figure 3:**
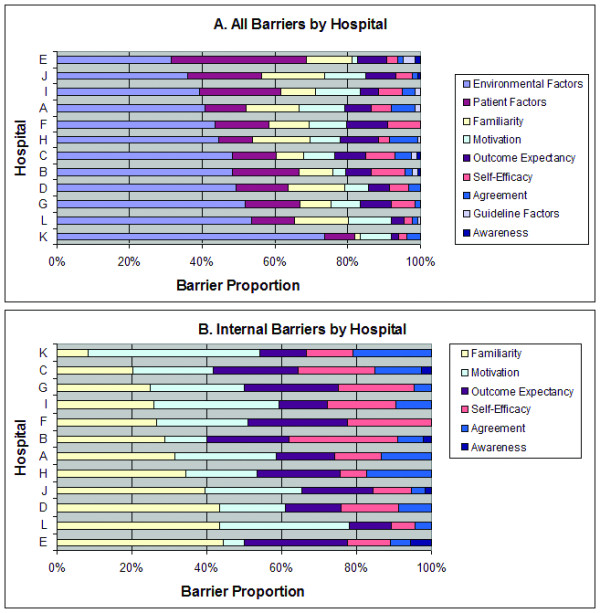
**Distribution of cited barriers by individual hospital**. Overall, the dominant barriers reported were external barriers and patient related factors.

Examples of responses which are illustrative of important internal barriers are provided in Table 4. The quotations were edited for readability but no substantive changes were made. Text added for clarity has been placed in brackets. When considering the internal barriers, nine of twelve hospitals cited guideline familiarity as most important (see Figure 3). Additionally, for eight of twelve hospitals, the top three cited barriers were guideline familiarity, provider motivation, and provider outcome expectancy. In contrast, lack of agreement with guidelines and lack of awareness of the presence of guidelines were the least important barriers for ten of the twelve hospitals.

**Table 4:** Barriers Internal to the Individual Provider

Barrier	Type of Participant	Representative response
Lack of Guideline Agreement	Emergency Physician	"They were rightfully upset when suddenly, based on one study [NINDS], when you had five previous studies that...had bad outcomes. Three of them they stopped early because of the bad outcomes. And yet here we were asked to change our therapy based on this one study."
	Emergency Physician	"A lot of it has to do with how much influence certain big-shots in emergency medicine have. There are some - one in particular who practices at Hospital X, just 10 miles down the road, he's been very outspoken against the use of tPA. And if you ever go to the national [emergency medicine] meetings and listen to these...docs speak, they can be very convincing. And I think that has had some influence on some people."
Lack of Guideline Awareness	Neurologist	"... when the patient goes to the neuroscience unit, and their blood pressure goes out of the parameters, I mean they don't initially call the neurologist, ...usually it's the family medicine resident. Unless the neurologist has specifically written something else."
		
Lack of Guideline Familiarity	Emergency Physician	"Did you say, 20 percent of patients that received placebo [in the NINDS trial] die? Twenty percent? That's impossible."
		
Lack of Outcome Expectancy	Emergency Physician	"And I have used it probably three times, and I've really been unable in the emergency department to see any significant improvement. I don't think I've had any complications, but oftentimes I don't get much feedback on how my patients do later on, so I'm not really sure how they did."
Lack of Self-Efficacy	Emergency Physician	"...some physicians are less comfortable with the whole process. You know, [some physicians would] explain risk-benefits to families, and [would not be] giving the lytics without prior discussion with the neurologist, or some other ER physicians would be comfortable without ever talking to neurologists, and doing everything and then just coordinating care with the intensivists."
		
Lack of Motivation	Emergency Nurse	"And they'll go back in there and double-check that patient seven times in order to say, oh, they're improving, you know, as one of the relative contraindications... Their stroke scale score was 14 and now it's 12, so they're improving-- we don't have to give it. You know. Whew! That kind of a thing."

Representative responses from participants that were coded as barriers. All internal barrier types are included.

The external barriers of environmental factors and patient factors dominated the barriers discussed for every hospital (Figure 3a) and for all participant types. A great deal of discussion focused on the environmental (or systems based) barrier of radiology, particularly regarding failure of adequate communication of the time sensitive nature of computed tomography (CT) ordering and interpretation. Interestingly, radiologists in some cases also discussed the lack of a specific process to alert them to the emergent nature of these CT scans. The limited availability of neurology was frequently discussed as well. In some areas this was a general lack of neurologists and in others it reflected a lack of evening/weekend coverage. Fear of liability both for giving and not giving tPA also received moderate attention as an external barrier.

**Figure 4:**
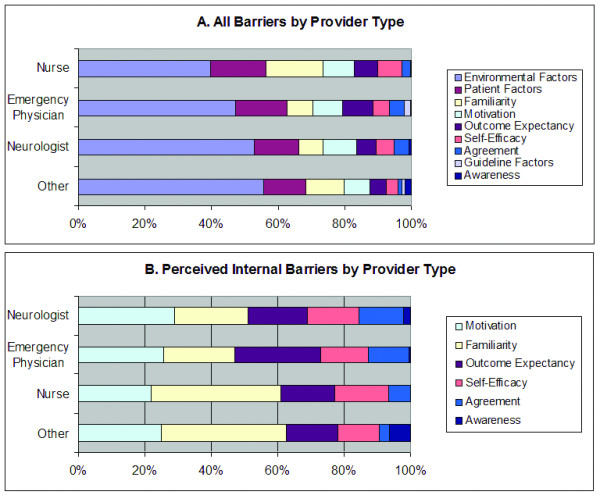
**Distribution of cited barriers by acute stroke care provider type**. In general, nurses perceived lack of guideline familiarity as the biggest barrier whereas physicians (both EM and neurologists) perceived physician motivation as the primary barrier.

Within the internal barriers (Figure 3b), most participants identified lack of guideline familiarity as a large component of their hospital's barriers. Most also had either outcome expectancy or motivation as an important barrier. The lack of self-efficacy appeared to be an important contributing barrier in several hospitals as well. When considering barriers organized by type of provider, the external barriers of environment and patient-controlled factors again dominated the perceived barriers (see Figure 4a). Regarding the internal barriers, nurses perceived lack of guideline familiarity as the most important barrier whereas physicians (both EPs and neurologists) perceived physician motivation as the primary barrier (see Figure 4b). Of the barriers defined as internal to physicians, the most important were familiarity with and motivation to adhere to the guidelines, self-efficacy, and outcome expectancy.

## Discussion

We investigated barriers to the provision of thrombolysis for stroke in a randomly selected group of community hospitals, focusing on the beliefs and experiences of health care providers most likely to be involved in providing acute stroke care. In general, almost half of the discussed barriers were considered external to the provider (e.g., systems issues, patient delays). The barriers internal to the provider were prioritized according to a model of physician behavior change[9].

The hospital barrier assessment process was important because although the top barriers were similar across sites, there was still inter-site variability. This stresses the need for interventions targeted to individual hospital and provider barriers. The optimal and efficient design of interventions to improve health processes requires a firm understanding of the knowledge and attitudes of the group targeted for the intervention[17]. This is analogous to establishing understanding of the pathophysiology and course of disease prior to the development of a treatment.

Past work on barriers to thrombolysis has focused on patient- or hospital-level characteristics and not provider-level barriers[4,6]. Our focus centered on the knowledge and attitudes of the providers at the bedside who were deciding whether and how to offer thrombolysis to stroke patients. Providers were cognizant of the importance of delayed presentation and the difficulties inherent in patient and family symptom recognition and often reported these as important barriers. Further work on the exploration of the interaction between the physician offering therapy and the patient or family member deciding on receiving therapy could provide additional insights into improving treatment rates.

The finding that EPs frequently cited lack of motivation to adhere to, and lack of familiarity with, the content of guidelines for stroke thrombolysis is intuitive. An example of this is the observation that physicians will repeatedly examine patients looking for improvement to justify withholding treatment. Prior and current ongoing methods of examining thrombolytic utilization and eligibility have not captured this. Designing interventions that recognize treatment should occur promptly to combat this hesitancy is important, particularly since providers may delay even further with the recent publication of data that potentially expands the time window for thrombolysis[18,19]. The lack of familiarity with the guidelines cited as a barrier by many respondents often focused on specific procedural issues such as blood pressure control. Difficulty with these and other aspects of the post-treatment guidelines have been observed in prior cohorts of thrombolytic treated stroke patients, although prior investigations focusing on clinician failure to treat patients meeting criteria have been limited[20]. The qualitative methodology utilized in the current investigation was crucial to identifying these important issues and others that have not been captured by prior reviews of thrombolytic cases or EP surveys.

The relative minimal importance ascribed to a lack of agreement with the guidelines is surprising, especially in light of the past controversy that stroke thrombolysis has generated within the field of emergency medicine[21,22]. This implies that changing physician practice with regard to stroke thrombolysis may not require changing minds, per se. Instead, increasing physician familiarity, confidence (self-efficacy), and motivation to deliver the treatment are likely to be of higher yield. Further investigation of the limited guideline disagreement perceived by EPs will be needed. Further conclusions on this topic may be facilitated through quantitative survey data. In addition, a small number of hospitals seemed to have clusters of higher perceived guideline disagreement. This suggests that clustering within physician groups is an important consideration for evaluating and improving barriers to care.

Our separate interviews with nurses and EPs provided unique findings. The repeated re-examination phenomenon was described by emergency department nurses. This specific example typifies the perceived barrier that was cited as most important by many nurses: lack of motivation. The picture that is painted is that of the clinician who is uncomfortable and unsure when faced with the potential of having to administer a thrombolytic agent. It is doubtful if this barrier would have been articulated as clearly without interviews restricted to individual provider types.

This work has several important limitations. We did not generally seek "saturation" by performing repeat focus groups with the intent of further delving more deeply into specific themes. We used an existing taxonomy to classify responses, which might have missed barriers that did not fit well into any of the categories. The integration of these results with quantitative methods and overall response to the targeted educational interventions (as evidenced by change in tPA treatment rates), is not possible at this point in the overall trial. We focused only on 12 hospitals within Michigan, and while these hospitals came from diverse geographic and socioeconomic areas, these findings may not be widely generalizable. There is a potential that participants in the focus groups and interviews were generally more positive towards stroke thrombolysis, although it is also plausible that participants with strong negative opinions would also be extremely motivated to participate. Overall it appears that a range of opinions were represented by our participants. This contributes to the richness of the findings of the current investigation.

## Conclusions

In summary, healthcare providers responsible for acute stroke treatment perceive environmental and patient factors as the most important barriers to adherence with the AHA acute stroke guidelines. With respect to internal barriers, nurses perceived lack of guideline familiarity as the biggest barrier whereas physicians (both EPs and neurologists) perceived physician motivation as the primary barrier. Overall, the minimal discussion of lack of physician agreement as a barrier is interesting in light of ongoing controversy over the use of tPA for stroke in the field of emergency medicine. Greater knowledge of the barriers which impede the widespread adoption of acute stroke thrombolysis is crucial to designing effective educational interventions to improve guideline adherence and may be informative in other areas where difficult risk/reward decisions are made on an emergent basis.

## Competing interests

The authors declare that they have no competing interests.

## Authors' contributions

PAS conceived, obtained funding, and supervised this study. WJM developed the analysis and data collection methods. WJM, SAF AMS, and PAS all participated in data collection. WJM and JJM performed the data analysis. WJM wrote the first draft of the paper; all authors have read and edited the paper for content and approve of this manuscript. WJM and JJM have full access to all of the data in this study and take responsibility for the integrity of the data and the accuracy of the data analysis.

## Pre-publication history

The pre-publication history for this paper can be accessed here:

http://www.biomedcentral.com/1471-227X/11/5/prepub

## Supplementary Material

Additional file 1 **This is the final focus group script that was used for emergency physician or nurse focus groups**. Click here for file

Additional file 2 **This is the coding guide developed by the investigators with conventions used in assigning themes**. Click here for file
