# Supplementary material for: Provider perceptions of barriers to the emergency use of tPA for Acute Ischemic Stroke: A qualitative study
Source: BMC Emerg Med. 2011 May 6;11:5. doi: 10.1186/1471-227X-11-5 (PMC3112102; doi:10.1186/1471-227X-11-5)
Supplement: Additional file 1 — This is the final focus group script that was used for emergency physician or nurse focus groups. [file 1471-227X-11-5-S1.DOC]

**Focus Group Discussion Guide Version 3**

Time 0:00

Process Overview: (5 **minutes total)**

**Introduction:** **(2 minute)**

“The INSTINCT trial is a randomized controlled study investigating the effect of a comprehensive barrier assessment and targeted educational intervention on the appropriate use of tPA. Your hospital is one of the intervention sites. This focus group will help guide us as to which barriers to address first through the educational interventions and will also help us to improve the educational interventions.

As you know, the goal of our study is to test whether we can increase the use of tpa for acute ischemic stroke by implementing a variety of behavioral interventions (such as education, system changes, resources). To do this effectively, it is important that we understand how you feel about tpa use, how often tpa is currently used, what things in your environment support you in using it and what barriers might exist. Every institution has issues and people that you could consider to be a barrier. To address these effectively, it’s very important we take a neutral approach. So nothing is “right or wrong”, “good or bad”.

“Everything that you discuss in this session will be confidential and your name will not be associated with any specific comments that you make. We are recording this session only so that we can review it later to identify specific themes or comments that will help us develop our plan.

While it is certainly acceptable to discuss particular areas of acute stroke care where you feel your institution excels, the major focus today is to identify barriers – so that we can target areas needing improvement. This may leave you feeling that we have focused too much on the negative – don’t worry – there will be other opportunities to highlight the successes at your site”

**Ground Rules / Etc. (1-2 minutes)**

Please be as open as possible

Please mention any barrier that you may find important even if you are not in agreement with others

Everyone’s opinions are important. There is no need to convince others to see your point of view.

Please do not interrupt others – as a courtesy to them and to our transcriptionists!

Try to speak towards the nearest microphone

Be yourself, Be open, Be honest (and blunt), Listen, Speak

**Prior to phase 1. Consent forms (1-2 minutes)**

Do this prior to starting the recording

***“Before we can begin, we need to obtain your consent to participate. Please review this document and sign on page 2. We will provide you with a copy of the signed consent before we leave today.”***

“Does anyone have any questions about the consent forms? Please be sure to sign them and turn them in now.”

*Try to identify people by name when asking questions and transitioning. i.e. “That was a very interesting comment, John, tell us more.” Do not do this so much that it becomes un-natural or obtrusive.*

*Areas below that are highlighted represent aspects of compound questions – you should ask the first question and then pause to allow the group to respond. Use the highlighted questions to go deeper into the responses or to address issues that were brought up by the group.*

**For late arrivals – state the following – “Hello, thank you for coming; we’re talking about (current topic area). Please review the consent form and if there are no problems with it then please sign it and jump right in. We will provide you with a copy today. If you have questions about the consent form let me know and I will address them when others are responding.”**

**0:05**

**TURN ON RECORDER**

**Phase 1. General questions (attitudes and past experiences) 36 minutes**

First, let’s have everyone introduce themselves. **1 minute** *During the nursing focus group add* “**and what your role is**” *to the end of question.*

Let’s talk about treating stroke and tpa use. Has anyone ever given tPA for stroke? If so, how did it go? How did you feel about it? “**5 minutes**

If you have not used tPA for stroke why do you think that is? **5 minutes**

What do you think the overall attitude is toward using tPA for stroke in your own ED? In general, who facilitates that belief system (you don’t have to name names) ? *(Probe : i.e. departments, administration)* **5 minutes**

**Physician Group Question (5 minutes):** Can you pretend that I am a 75 year old, who had the sudden onset of left sided weakness, so severe that I cannot lift my arm or leg, 1 hour ago and I have no contraindications to tPA treatment. Take me through the consent conversation you would have with me and or my family using the same language you would use in clinical practice.

**Nursing Group Question (5 minutes):** What are your perceptions regarding the consent process with regards to treatment with tPA for stroke? What do the physicians say and how is the information presented?

Overall, how do you believe your ED views acute stroke patients? Is it perceived as an emergency like an acute MI or trauma? Do they feel ownership of the treatment?

Are there differences in these views between ED physicians, nurses and consultants here at your institution? **5 minutes**

**Physician Group Question:** What is your interaction with neurology when caring for a stroke patient who is a candidate for tPA? (**5 minutes)**

**Nursing Group Question:** Why do you think Emergency Physicians are reluctant to treat stroke with tPA? **(5 minutes)**

Do physicians/nurses feel confident in their ability to identify patients eligible for tPA and in their ability to administer tPA at your hospital? **5 minutes**

**Try to give phase 1 more time**

**0:41**

**Phase 2. Focused questions on barriers to tPA use at your hospital (12 minutes)**

(*Refer* *to the flip chart for a schematic of the tpa process and its various stages*)

Sometimes it’s helpful to reflect on all of the various stages/steps in the process of giving tPA for acute stroke. Let’s go through them one at a time and see the barriers to tPA use in acute stroke that you foresee - or have experienced -? *It’s very important we hear from everyone.*

*Customize based on what you heard in phase 1. If it was widely covered in phase 1 then summarize and ask for additional information.*

Q. Lets start with Stage 1, **Pre-hospital** . Does anyone want to share their thoughts on barriers in this stage?

Probes

Do any of your EMS systems bypass other hospitals to come to you? Or vice versa?

Are you the only hospital in the area?

Q. How about Stage 2…Arrival at the **Door** - the initial ED evaluation?

Are there any triage barriers?

Probes

Do you use stroke assessment tools at triage? What about in the ED?

Overcrowding, availability of a room?

Time to physician evaluation?

Q. In Stage 3, collecting the clinical **DATA,** are there barriers to obtaining some elements in making tPA treatment decisions?

Probes

Adequate emergency department staffing?

Labs?

CT Scanning?

CT interpretation?

Establishing stroke onset time?

Patient monitoring

Blood pressure management?

**Physician Group Question**

Q. In Stage 4, making a final tPA treatment **DECISION**, are there things at your hospital that facilitate or hinder making a treatment decision?

Probes

Obtaining specialty consultation?

Discussion with the family?

Obtaining consent to treat?

**Nursing Group Question**

Q. In the 5^th^ Stage, delivering the **DRUG**, are there things at your hospital that facilitate or hinder tPA drug delivery in acute stroke:

Probes

Physician communication with nursing to administer drug?

Availability of drug in ED?

Dosing calculations and mixing?

Drug administration?

Q. In the final, 6^th^ Stage, **POST-TREATMENT**, what inpatient issues at your hospital facilitate or hinder tPA use?

Probes

Lack of critical care beds?

Lack of specialty consultation? (e.g. neurosurgery)

**0:53**

**Phase 3. Ranking (5 minutes)**

We will now go around and want everyone to state what they feel the one or two most important barriers to tPA use in eligible candidates at your hospital are and why? *“It is important that we get responses from everybody here”*

**Phase 4. Wrap up (2 minutes)**

“Do you think we have captured the major issues?”

“Can you think of anything we haven’t covered that we should know about?”

“So what happens next? We will review the recordings, categorize comments and include the data collected from this group in the overall barrier assessment for your hospital. We will use this data to structure and prioritize the educational program we will be soon bringing to your site.

“Thanks for all of your help”.

**Flip Chart A**

Overview of Focus Group

Phase 0. Introduction / Consent form

Phase 1. Attitudes and past experiences with acute stroke care

Phase 2. Barriers at age step in the acute stroke treatment schematic

Phase 3. Ranking of most important barrier

Phase 4. Summary and conclusions.

**Flip Chart B**

Acute Stroke Treatment Schematic

Stage 1 **PRE-HOSPITAL** care (**All)**

Stage 2 Arrival at the **DOOR** (**All)**

Stage 3 Collecting the **DATA** to make tPA treatment decisions (**All)**

Stage 4 Making the final tPA treatment **DECISION (Physicians)**

Stage 5 **DRUG** delivery **(Nurses)**

Stage 6 **POST-TREATMENT** care B(**All)**
