# Supplementary material for: Provider perceptions of barriers to the emergency use of tPA for Acute Ischemic Stroke: A qualitative study
Source: BMC Emerg Med. 2011 May 6;11:5. doi: 10.1186/1471-227X-11-5 (PMC3112102; doi:10.1186/1471-227X-11-5)
Supplement: Additional file 2 — This is the coding guide developed by the investigators with conventions used in assigning themes. [file 1471-227X-11-5-S2.DOC]

QUALITATIVE DATA ANALYSIS

THE INSTINCT TRIAL

INVESTIGATOR INSTRUCTIONS

VERSION 1.3

May 2007

# Contents

| Page |  |
| --- | --- |
| 2 | Contents |
| 3 | Introduction |
| 3 | Overview of qualitative data acquisition in trial |
| 3 | Theory utilized |
| 4 | Specific barrier types |
| 5 | Coding Process |
| 6 | Database use |
| 9 | How to code using NVivo 7 |
| 10 | Adding annotations |
| 11 | Summary |

# Introduction

Thank you for agreeing to participate in the qualitative data analysis of the INSTINCT trial. This document will provide instructions for the use of the NVivo 7 software package. In addition the methods to be used in the coding of the data will be explained. As qualitative research has some terminology that may be unfamiliar to you, we have provided a glossary of terms to assist.

The process by which the text will be analyzed and a general timeline of which elements will be ready for analysis when will be discussed.

If you have further questions please do not hesitate to contact me [wmeurer@med.umich.edu](mailto:wmeurer@med.umich.edu).

**Version 1.1**

June 27, 2007: After meetings between the two coders have occurred to discuss disagreements in coding of two of the initial focus group transcripts the instructions have been revised and several areas have been clarified. This guide will continue to be used by the coders and will incorporate an additional section for instructions on how to code the interview data from the site barrier assessments.

**Version 1.2**

June 29, 2007: Further changes from the above meeting were incorporated into the guidebook.

**Version 1.3**

July 11, 2007: Agreement was improved after the third comparison of focus groups. Some additional clarifications to this guide concerning guideline factors and motivation were made.

# Overview of Qualitative Data Acquisition in the Trial

We will be working with data from focus groups and structured interviews in this trial. Initially, six focus groups were conducted at the Champions Meeting in late March of 2007. Participants in these groups included emergency physicians, neurologists, administrators, hospitalists, pharmacists, nurses and others. A structured discussion guide was developed and used by the moderators of each of the focus groups. All of these focus groups have been transcribed and are the focus of the initial phase of analysis. This data has individual identifiers (hospital and participant role) hidden.

Each of the twelve intervention sites will have an on-site barrier assessment – this will include separate focus groups of emergency physicians and nurses, along with structured interviews of neurologists, administrators, and radiologists. These focus groups will be analyzed in the same fashion as the focus groups from the Champions Meeting. The interview guides for the in-depth interviews are being finalized, therefore additional instructions about the methods for analyzing this data will be provided in a subsequent version of this guide.

The data collected will be used to prioritize content for educational interventions at the sites. In addition, the data will provide the INSTINCT investigators with useful insights and real examples to be able to provide CME programs which are individualized to the specific sites.

# Theory Utilized

The methods we are using are described in greater detail in the November issue of Academic Emergency Medicine. Briefly, we will be using the framework set out by Cabana (JAMA 282:1458-1465) to characterize the types of barriers to delivering rt-PA for acute ischemic stroke that are discussed by participants in the focus groups and interviews.

The goal of the coding process is to attribute all barrier specific responses to one of the categories that we have pre-specified (discussed below.) Therefore for the initial coding of the data we will largely be performing a content analysis. There may be future studies in which the data on specific barriers types is further analyzed using a grounded theory approach and also analyzed by the differentials in barriers perceived and deemed important by different stakeholders. (For example – a study on the perceptions of ED nurses regarding physician level barriers.)

Knowledge

Attitudes

Behavior

Lack of

Familiarity

Lack of

Awareness

Lack of

Motivation

Lack of Outcome

Expectancy

Lack of

Agreement

Lack of

Self-Efficacy

Patient factors

Guideline

factors

Environmental

factors

**EXTERNAL**

**INTERNAL**

# Barrier Definitions

## Lack of Agreement

This barrier should be coded when the text relates to the respondent not agreeing with the guidelines. This can include but is not limited to personal interpretation of the evidence, applicability to specific patients, and lack of confidence in the guideline developer or the process by which the guideline was developed. This barrier should also be coded if a general lack of agreement with guidelines in general (i.e. “too cookbook”) is obverved. Also, if the respondent cites national or local opinion leaders who disagree with this guideline that would be most appropriately coded under this section. This category also includes being too liberal in treatment despite the presence of absolute contraindications to treatment (such as time.)

## Lack of Awareness

This barrier deals with physicians not being aware of the existence of guidelines for acute stroke care. This will likely be a relatively uncommon finding. In cases when the lack of awareness of others in the patient care team (i.e. inpatient team being unaware of guidelines regarding blood pressure management) that would be appropriate to include here and potentially also an environmental barrier as well if it appears to be a reflection of institutional politics or common practice. This does **NOT** include not knowing about the existence of stroke scales.

## Lack of Familiarity

The lack of knowledge of the contents of the guideline or the inability to properly access or apply the guideline should be coded here. This would include overuse or desire for overuse of tPA outside of the guidelines (i.e. feeling that a strict time window is not necessary to ensure safe treatment.)

This category is not meant to reflect a lack of familiarity with emergency care in general or with stroke patients in general. Certainly, if a respondent cites that they only see one eligible stroke patient every 5 years and do not recall all of the inclusion and exclusion criteria this would be logical to include here.

We have stated that reluctance to treat those at the extremes of age and at the extremes of severity is generally a lack of familiarity with the guidelines which do not include these as contraindications (other than very low severity and age less than 18 years.)

Physicians and nurses who fail to recognize stroke symptoms would be included here. (But not EMS providers – this would be included under environmental.)

## Lack of Outcome Expectancy

The physician believes that the performance of the guideline will not lead to the desired outcome or there is a prominent, stated fear of a bad outcome. Discussions regarding the lack of a “Lazarus effect” would be an example of this barrier.

## Lack of Self Efficacy

The respondent believes that they cannot perform the guideline recommendation correctly. This may be a reflection of personal experience or available resources. (Lack of available resources generally should be coded as an External Barrier – Environmental Factor.) It may also reflect a situation in which the physician or nurse does not feel that they are unable to treat the patient effectively with the tools they are given (i.e. given that there was a vague reading from radiology it is hard to confidently offer this therapy.)

## Lack of Motivation

Inertia can be a powerful force. This barrier should be coded when the discussion includes the difficulty in changing clinician habit and routines. This should also be coded when it appears that there is “reluctance” to treat. Willingness to treat, whether “they (ED physicians) like tPA or not,” and the concept of “physician perception would reflect a lack of motivation to comply with the guideline.

## External Barriers – Environmental

This is a large category. It encompasses the environment in which care is delivered. It includes lack of resources, institutional hurdles, lack of consultants, lack of reimbursement, and of special importance in acute stroke care – liability. In acute stroke care, pre-hospital, triage and overcrowding issues also fall into this category. Geography generally should be included here. Inpatient floor and nursing home issues should generally be included here.

## External Barriers – Patient (and Family) Factors

Patient may present with a desire to get the drug. Patients may also present problems based on their own failure to recognize stroke symptoms or present in a timely fashion. Family preferences and difficulty in finding family would be a patient factor. Difficulty in communication due to language barriers would also be a patient factor. Delayed presentation due to geography would usually be an environmental factor; however if the family decides to drive the patient instead of activating EMS this would qualify as a Patient level barrier. If the patient chooses an inappropriate level of care for their symptoms (i.e. presenting to an urgent care center with a dense hemiparesis) that would qualify as a patient factor; however if EMS and the urgent care center cannot promptly move that patient to a facility with an appropriate level of care that would then generally be an environmental barrier.

## External Barriers – Guideline Factors

The characteristics of the guideline itself can present a barrier. The presence of contradictory guidelines or “position statements” would fall into this category. This includes lack of confidence in the guideline, the body or bodies which create the guideline, and the guideline development process. If the guidelines are not felt to be clear this would also be in this category.

# Coding Process

The first step will be the coding of all of the Champions Meeting focus groups to one of the 9 categories (or nodes) specified above. Not all text from the transcript should be attributed to one of these categories. You should not code any of the text from the moderator. In addition, if the respondent is speaking about a strength and not a barrier then this should also not be coded.

The area to be coded should generally be the entire paragraph associated with the speaker’s response. If you code a bit of the extra empty space after the response this could potentially contribute to a lack of inter-rater agreement later when this is checked using the software. In the event there are several short paragraphs that are closely linked and provide the “story” of that response they can be coded as a group.

*However – the coding unit for this analysis has been prospectively defined as the paragraph. In some cases there may be a great deal of cross talk may be present and the small chunks of text that remain may not make a great deal of sense on their own. In general, if the coding unit (the paragraph of speech from a single respondent – which in practice may be a few words only) cannot stand on its own or does not clearly reference a predetermined barrier than it is best left* ***UNCODED.***

After the initial coding is performed, the agreement will be checked. If it is not within 80% between the investigators they will have a meeting and discuss the areas not in agreement and attempt to reach a consensus. If there is continued disagreement, the PI for the study will adjudicate. At this point the PI will receive a report summarizing passages in which there is disagreement along with a copy of this manual and will then meet with the coders to improve the manual and provide additional guidance as to how areas of difficulty should be treated.

Any changes that are felt to be required to the codebook for the barrier assessment phase will be introduced at this time and the previously coded areas will be revised to reflect these changes.

**If a response is not directly a barrier to rt-PA use but appears to be a barrier to delivering quality acute stroke care then you should code this text using one of the existing categories or nodes.**

# Glossary of Important Terms

**Attribute –** Descriptive characteristics of participants (such as role or hospital)

**Case –** This is a node that describes a particular respondent – such as the moderator or an ED physician from hospital X.

**Code -** The process of assigning data from source documents to specific categories or nodes.

**Coding stripe -**  A useful tool to provide an ongoing color record of the nodes which have been coded.

**Node -** The category that can be assigned to a section of text. Nodes can have sub-nodes (or child nodes). A node that spawns other nodes is a parent node. In the program these are listed under “Tree Nodes.” In the initial coding phase of this study the only true Tree Nodes are the three that fall under External Barriers – environmental, guideline factors, and patient factors. However all nodes currently used in the codebook are under the tree nodes as it is likely the data will be more deeply divided into additional child nodes in the future (i.e. – fear of liability as a child node of External Barriers – Environmental Factors)

**References -** Sections of text which have been coded

**Sources -** A document, such as a focus group or interview transcript.

# Database Use

The text will already be coded for the “cases” when you will start to use it. This means all the text will be attributed to one of the participants or the moderator (or coded as un-attributable). You should open the file titled – Name Qualitative Database.nvp – this can be opened from My Computer or by opening the NVivo program first.

Next you will choose a Source (or document) to code first. This may be done by clicking on Sources in the lower left corner.


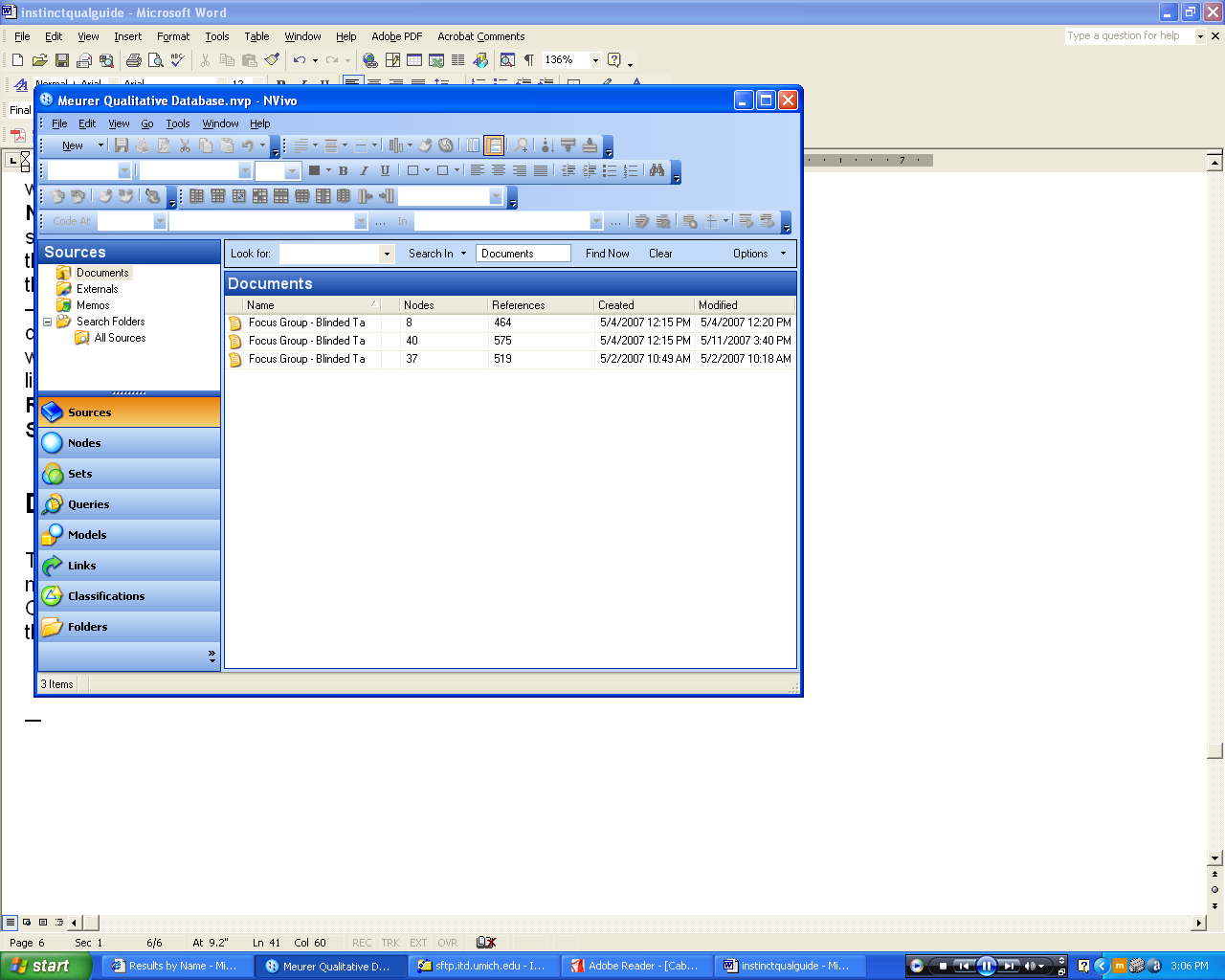


This will bring up the list of documents. You should double click on one to open it.

It will initially open in a lower window in the screen.


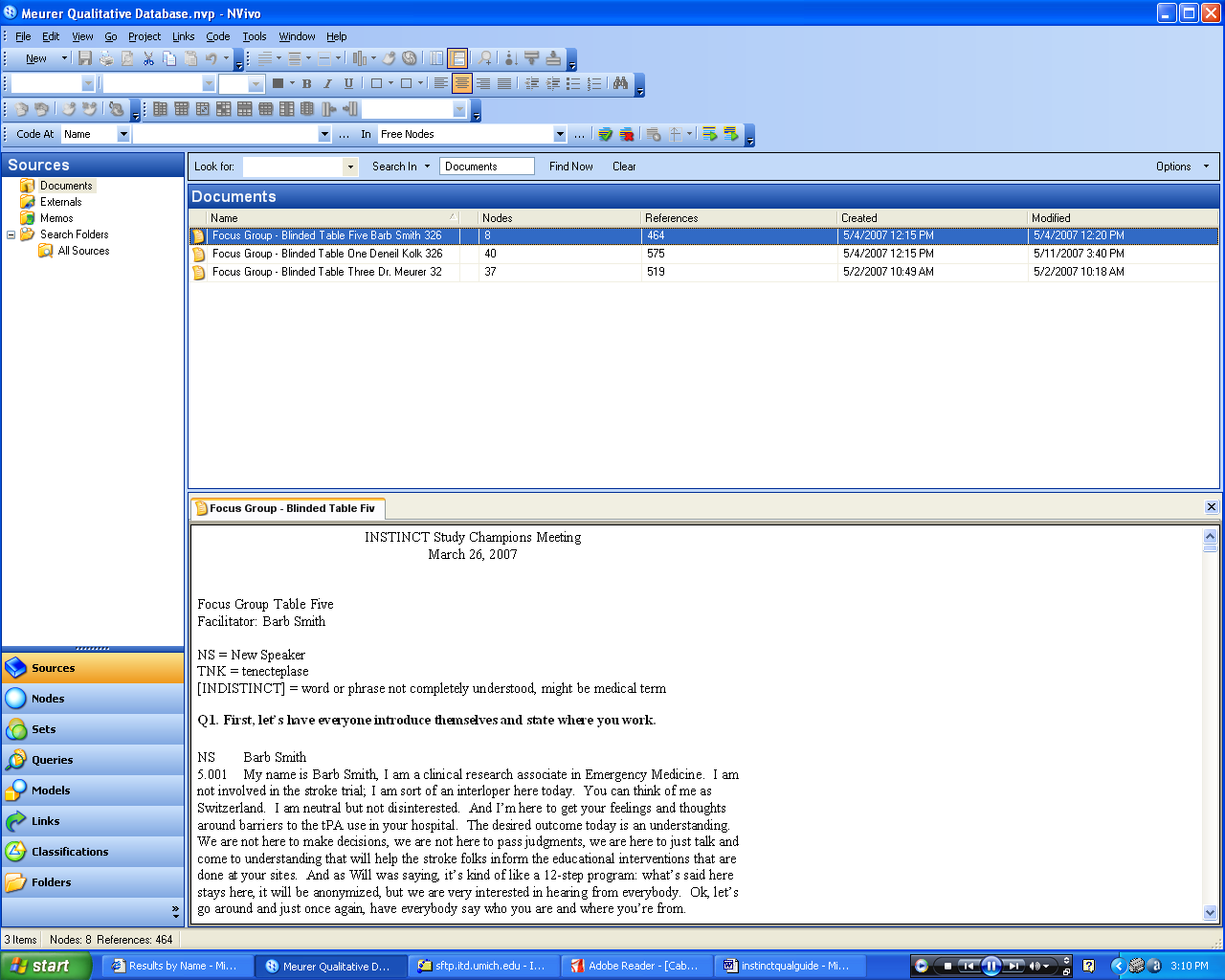


You may click on Window from the menu bar and select the choice “Undock: All.”

This will move the document into its own window which will increase the effective size you have to work with.

You may now click on View from the menu bar and select Coding Stripe. You may select “Show Nodes Most Coding Item” for now. This will show you all the attributions to sources currently because you have not yet assigned any codes. Later when you have coded some of the text you can use “Show Nodes Coding Item” to select which of the tree nodes you wish to display. Currently the program will not allow you to do this as you have not assigned any of those nodes to this “source” or document yet.

The program does NOT have any autosave feature; therefore you should manually save your work frequently. The program will prompt you to do this every 15 minutes.

#
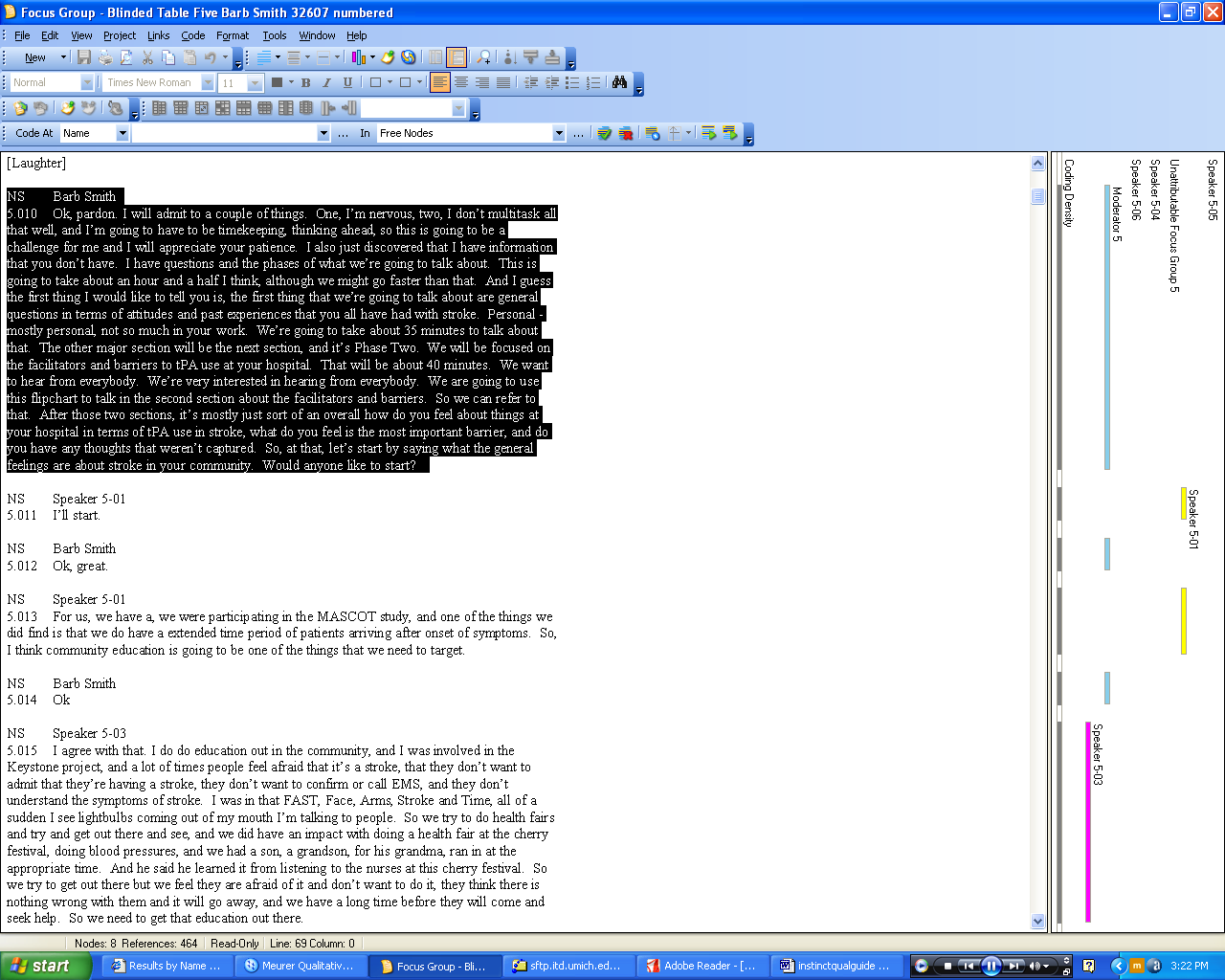
How to code using NVivo 7

Within the document you are working with you should highlight the text you wish to code. Again, to improve reliability and standardize the coding process you should select whole paragraphs (even if they are very long) when doing this work. You may need to code for several different barriers in this paragraph and that should all be done at once. As an example – the first 2-3 sentences may deal with lack of outcome expectancy and the last 2-3 deal with environmental factors. The whole paragraph should be coded for both.

After you have selected the text that you wish to code then you can right click. Then select code and then select “Code Selection at Existing Nodes.” Never select Code Source at Existing Sources (this will code the entire document with whatever node you are working with.) This will bring up the following screen.


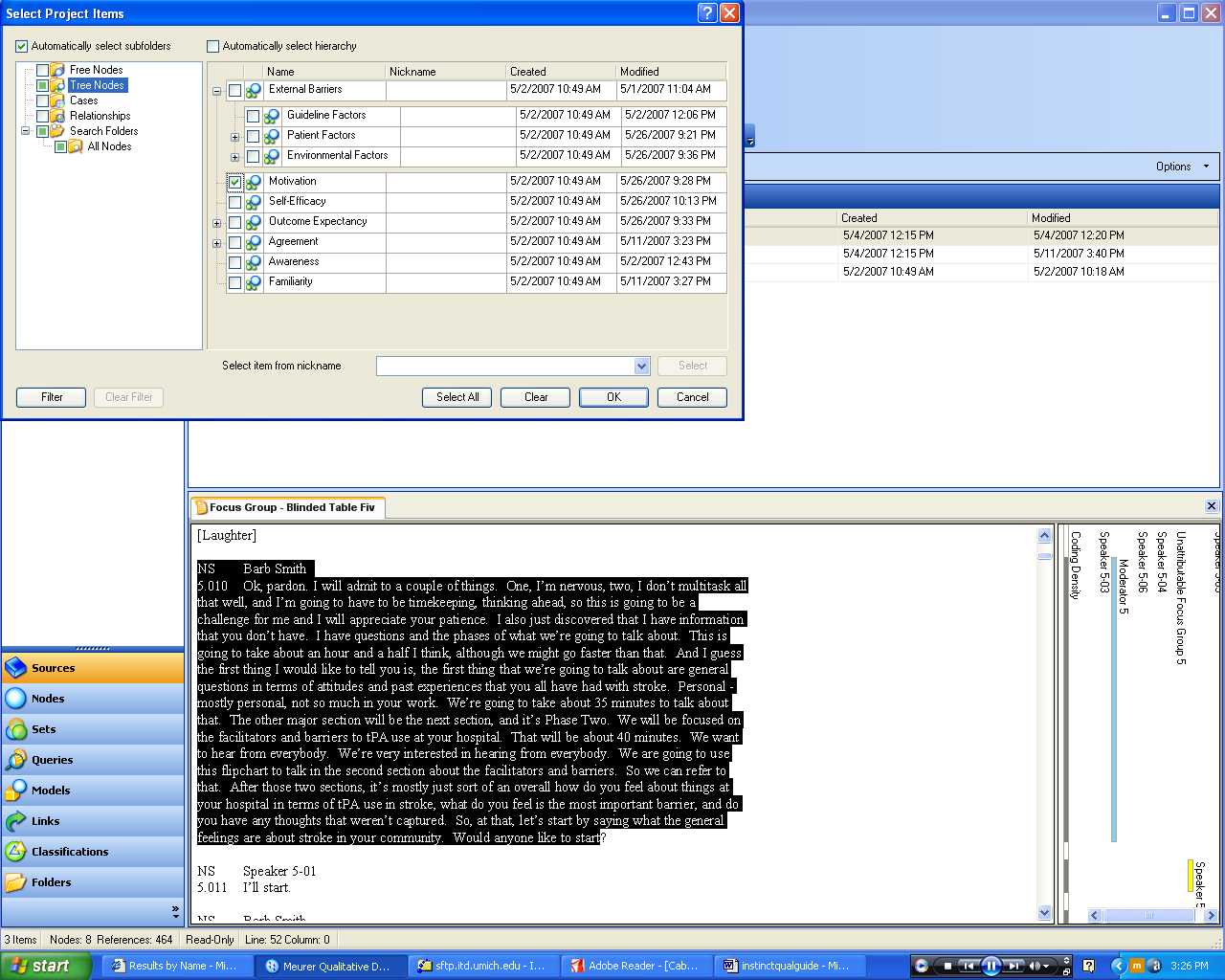


As you can see – Motivation is already selected. Unfortunately, every time you open up the coding window it populates the same choices as you most recently coded. I would recommend that every time you open up this window, **before you do anything else – CLICK ON “CLEAR.”** This will guarantee that you are only selecting the nodes you really want to code for a particular section of text. You will need to click on Tree Nodes on the left to bring up the choices. (In your version only the External Barriers tree node should have a plus and you will need to click that plus to expand to see the Guideline Factors, Patient Factors, and Environmental Factors.) After you have selected all of the nodes you wish to associate with this area of text then you can click on OK.

If you made a mistake and coded the wrong nodes and you recognize it right away you can click on Edit on the menu bar and then choose “Undo.”

If you made a mistake and only notice it later you can select an area of text, right click and select “Uncode.” As its name implies, this will work the same as coding only it removes coding. It also is subject to the same quirk. **YOU MUST CLICK ON CLEAR** when you bring up the screen unless you are very sure that only what you want is selected.

#
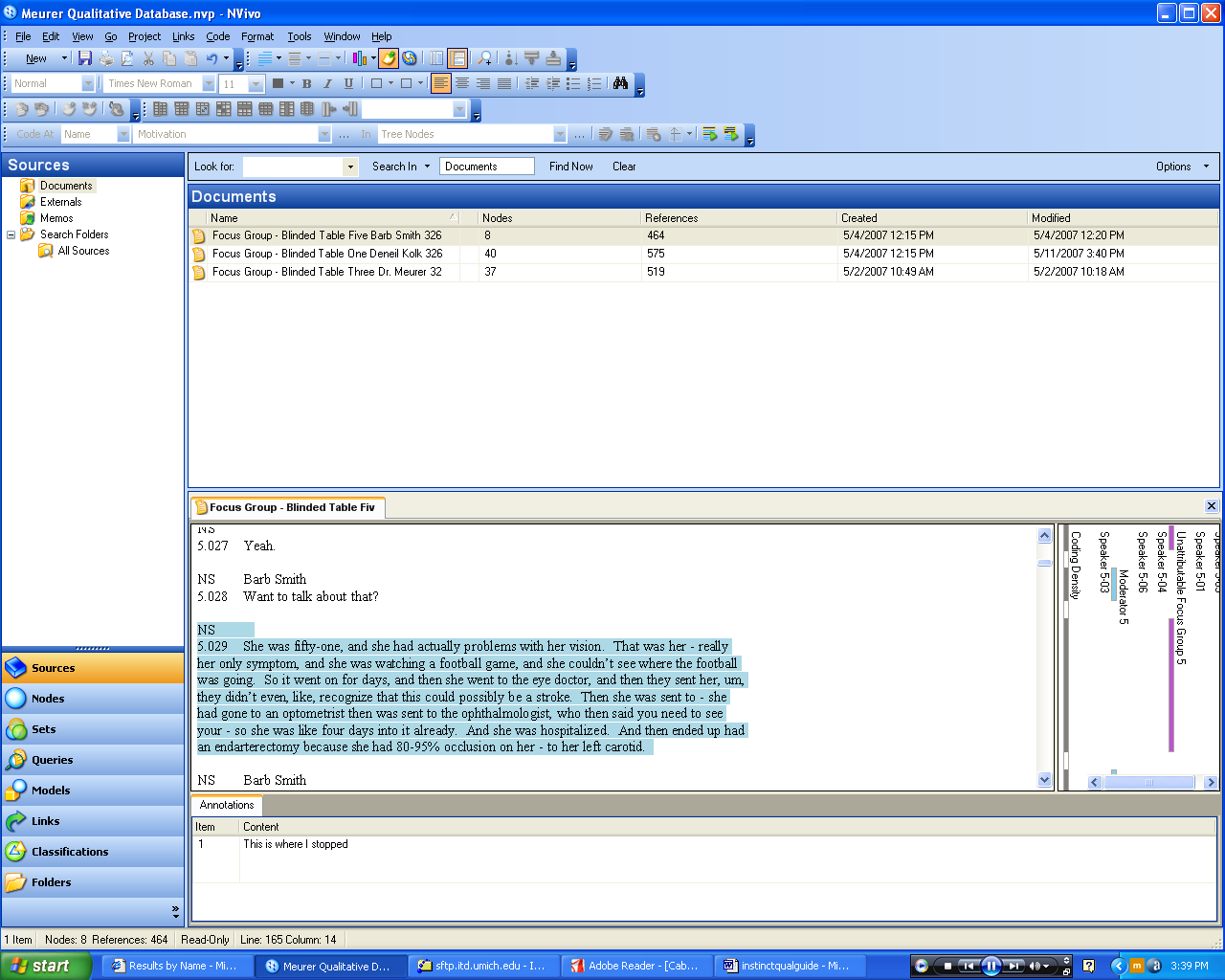
Adding Annotations

There may be a specific area of text you wish to make a note to yourself about – or you may want to mark it as it may represent an area that you think could benefit from being attributed to a new node or child-node. You may also want a place to mark your work so that you can pick up where you left off. (Each response in the transcript is numbered – so you could copy this down as well to remember your place.)

To place an annotation – select some text and then select “Links” from the Menu Bar. Select annotations and then click in the box at the bottom and add your text. (In this case – “This is where I stopped.”) The source text will become highlighted.

If you come back to the program and cannot see your annotation click on View from the Menu Bar and select “Annotations.”

You can right click in the box with the annotations and select delete if you want to eliminate it.

# Summary

The use of the NVivo 7 software can accelerate and expedite the process of performing qualitative data analysis. The software cannot however examine the text for the underlying themes and relationships in the way a human investigator can. The investigators in the trial sincerely appreciate the time and effort you are putting into this project. There are many other aspects to this software and it does have additional functionality – however we have attempted to provide the minimum “need to know information in this guide.

If you have questions about the process or the use of this software please contact William Meurer, MD at [wmeurer@med.umich.edu](mailto:wmeurer@med.umich.edu). He would also appreciate any feedback about these instructions so that they could potentially be improved for the future.

The future directions in which the investigators may utilize this rich repository of data may involve the further categorization of each of the types of barriers to provide models which lead to deeper understanding of the barriers to delivering rt-PA as part of acute stroke care.
